# A Sarsa(**λ**)-Based Control Model for Real-Time Traffic Light Coordination

**DOI:** 10.1155/2014/759097

**Published:** 2014-01-23

**Authors:** Xiaoke Zhou, Fei Zhu, Quan Liu, Yuchen Fu, Wei Huang

**Affiliations:** School of Computer Science and Technology, Soochow University, Shizi Street No. 1, Suzhou, Jiangsu 215006, China

## Abstract

Traffic problems often occur due to the traffic demands by the outnumbered vehicles on road. Maximizing traffic flow and minimizing the average waiting time are the goals of intelligent traffic control. Each junction wants to get larger traffic flow. During the course, junctions form a policy of coordination as well as constraints for adjacent junctions to maximize their own interests. A good traffic signal timing policy is helpful to solve the problem. However, as there are so many factors that can affect the traffic control model, it is difficult to find the optimal solution. The disability of traffic light controllers to learn from past experiences caused them to be unable to adaptively fit dynamic changes of traffic flow. Considering dynamic characteristics of the actual traffic environment, reinforcement learning algorithm based traffic control approach can be applied to get optimal scheduling policy. The proposed Sarsa(**λ**)-based real-time traffic control optimization model can maintain the traffic signal timing policy more effectively. The Sarsa(**λ**)-based model gains traffic cost of the vehicle, which considers delay time, the number of waiting vehicles, and the integrated saturation from its experiences to learn and determine the optimal actions. The experiment results show an inspiring improvement in traffic control, indicating the proposed model is capable of facilitating real-time dynamic traffic control.

## 1. Introduction

In most major cities, hundreds of thousands of vehicles distribute in a large and board area. It is a tough and complex work for us to effectively deal with such a large-scale, dynamic, and distributed system with a high degree of uncertainty [1]. Apart from the increasing number of vehicles in urban area, the fact that most of present traffic control systems have not taken full advantage of intelligent control of traffic light is one of the most important one [2]. People [3] have found that reasonable traffic control and improving the utilization efficiency of roads is an effective and economical way to solve the urban traffic problem for most cities. Traffic signal lights control policy, the most important part of intelligent transportation system, turns out to be even more essential [4].

However, as there are so many factors that affect the traffic lights control, off-line control policy model is not suitable for sudden and sporadic characteristics of road. Hereby, in this paper, we propose an online traffic control model which is based on Sarsa(*λ*) [5]. In our model, several traffic signal control modes are treated as candidate action selections; the vehicle speed and saturation of an intersection are viewed as context of environment, and common signal control indicators, including delay time, the number of waiting vehicles, and the integrated saturation are defined as return. In the experiments, the proposed model showed its ability to facilitate real-time traffic control.

## 2. Related Work

At present, the traffic control systems can be classified into static traffic control systems and dynamic traffic control systems, where the former often uses statistical approached to optimize the settings while the latter can adjust traffic controller duration dynamically according to real-time traffic conditions.

Many achievements in collaborative traffic flow guidance and control strategy have been made. The F-B method [6] has been widely used by many researchers and engineers of the transportation industry. By using the approach, the traffic jam problem was partly solved. Thereafter, there came many improved approaches [7] based on the F-B method. Driving compensation coefficient, along with delay time, was used to evaluate the efficiency of time allocation scheme [8]. The model minimized delay of waiting time, making the approach appear to be acute and reasonable. However, as the model could hardly deal with heavy traffic, we still need to find a more suitable approach.

The ability of intelligent traffic control as a good solution to traffic congestion problem has gradually received more and more attention [9]. However, congestion problems between adjacent intersections still need more efforts. The regional coordination control proved to be a good solution to this problem [10]. Although many area coordinated control methods were proposed, few of them yielded good results due to the lack of a clear regional control mathematical model, especially in complex environment with heavy traffic. However, due to the complexity and changeability, it is of little possibility to build an accurate mathematical model for traffic system in advance [11].

It has become a trend to solve traffic problems by taking advantage of computing technology and machine intelligence [12]. Among many machine learning approaches, reinforcement learning is suitable for the optimal control of the transportation system strategy as it does not require mathematical models of the external environment [13]. The study using the *Q*-learning algorithm [14] achieved online traffic control. The approach was able to choose the optimal coordination model under different traffic conditions. Some applications [15] that utilize *Q*-learning algorithm have received much significant effect. A paper implemented an online traffic control through *Q*-learning algorithm, yielding good effort in the normal state of traffic congestion [16].

## 3. Traffic Evaluation Indicators

Signal lights control plays a very important role in traffic management. A reasonable and good semaphores time allocation scheme guarantees that under normal circumstances the traffic moves smoothly. Frequently used traffic efficiency evaluation indicators [17] include delay time, the number of waiting vehicles, and intersection saturation.

### 3.1. Delay Time

The indicator delay time refers to the delay between the actual time and theoretically computational time for a vehicle to pass an intersection. In practice, we can get total delay time during a certain period of time and average delay time of a cross to evaluate the time difference. The more delay time represents the slower average speed of a vehicle to pass an intersection.

### 3.2. Number of Waiting Vehicles

The number of waiting vehicles shows how many vehicles are waiting behind stop line to pass the road intersection. The indicator [17] is used to measure the smooth degree of road as well as the road traffic flow. It is defined as

(1) $$\begin{array}{c} \text{wait}=\text{wai}{\text{t}}_{\text{G}}+\text{wai}{\text{t}}_{\text{R}}, \end{array}$$

where wait_G_ is the number of waiting vehicles before the green light and wait_R_ is the number of waiting vehicles before the red light.

### 3.3. Intersection Saturation

The indicator intersection saturation denotes the ratio of the actual traffic flow to the maximum available traffic flow. Intersection saturation is calculated as

(2) $$\begin{array}{c} S=\frac{\text{traffic}\;\text{flow}}{\left(\text{dr}*\text{sf}\right)}, \end{array}$$

where dr is the ratio of red light duration to green light duration and sf is saturation flow of the intersection.

### 3.4. Traffic Flow Capacity

Traffic flow capacity represents the maximum possible number of vehicles passing through the intersection. The indicator reflects effect of signal control strategy. We can see that traffic flow capacity is related to traffic signal duration. A longer passing duration generally yields a stronger passing capacity.

## 4. Temporal Difference Learning

Reinforcement learning is a framework to learn directly from the interaction and thereby achieve goals [13, 18]. Reinforcement learning framework is abstract and flexible and can be applied in many different applications.

In artificial intelligence field, agent is defined as an entity that has cognitive skills, the ability to solve the problem, and the ability to communicate with the outside environment. By agent, we can establish some system for controlling model. In fact, the model based on agent is an anthropomorphic model; as a result, we can control the behavior of people in the system and unify other control units, providing a unified description of the method. Agents are connected through network; agents act as intelligent nodes on the network, therefore constructing a distributed multiagent system.

**Figure 1:**
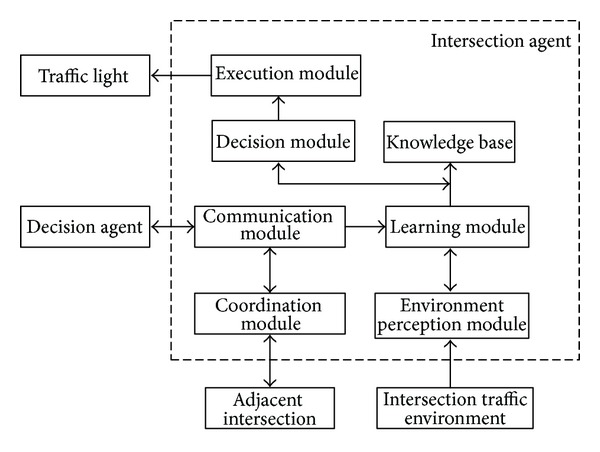
The agent model of intersection, including environment perception module, learning module, decision module, execution module, knowledge base, communication module, and coordination module.

The agent model of intersection is as Figure 1, including environment perception module, learning module, decision module, execution module, knowledge base, communication module, and coordination module.

**Figure 2:**
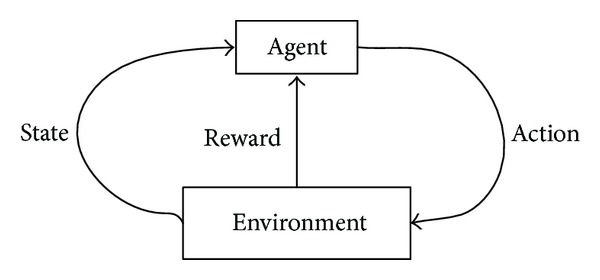
Framework of reinforcement learning. Agent selects an action; the environment responds to the action, generates new scenes to the agent, and then returns a reward.

In reinforcement learning framework, agent is a learner and decision-maker, interacting with environment which is everything outside of agent. Agent chooses an action; the environment responds to the action, generates new scenes to the agent, and then returns a reward. The framework [13, 18] of reinforcement learning is shown in Figure 2.

Agent interacts with the environment at each step during a discrete-time sequence (*t* = 0,1, …). At each time step *t*, agent gets the representation of environment denoted by state *s*_*t*_ ∈ *S*, where *S* is the set of all possible states; agent chooses an action *a*_*t*_ ∈ *A*(*s*_*t*_), where *a*_*t*_ ∈ *A*(*s*_*t*_) is all available actions. By taking the action, agent receives a reward *r*_*t*+1_ ∈ *R* and gets to a new status *s*_*t*+1_. The ultimate goal of agent is to maximize the sum of the rewards in long term. The mapping from state to action selection is policy of the agent, denoted by *π*_*t*_. Reinforcement learning solves how agent changes policy through experience.

The temporal difference (TD) learning is capable of learning directly from raw experience without determining dynamic model of environment in advance. Moreover, the model learned by temporal difference is updated by estimation which is based on part of learning rather than final results of the learning. These two characteristics of temporal difference make it particularly suitable for solving the prediction problems and control problems in real-time control applications. Given some experience with policy *π*, temporal difference learning updates estimated *V* of *V* ^*π*^ [19], as

(3) $$\begin{array}{c} V\left(s_{t}\right)⟵V\left(s_{t}\right)+\alpha \left[R_{t}-V\left(s_{t}\right)\right], \end{array}$$

where *R*_*t*_ is actual return after time step *t* and *α* is a step size parameter. Temporal difference learning updates *V* in step *t* + 1 using the observed reward *r*_*t*+1_ and estimated *V*(*S*_*t*+1_).

Let *Q* ^*π*^(*s*, *a*) be the value of taking action *a*, in *S* under a policy. *Q* ^*π*^(*s*, *a*) [20] can be defined as

(4) Qπ(s,a)=Eπ{Rt ∣ st=s,at=a}=Eπ{∑k=0∞γkrt+k+1 ∣ st=s,at=a}.

Sarsa (state-action-return-state-action) is an on-line TD control method. Sarsa(*λ*) is an eligibility trace [21] version of Sarsa. The update of *Q* ^*π*^(*s*, *a*) [22] depends on

(5) $$\begin{array}{c} Q_{t+1}\left(s,a\right)=Q_{t}\left(s,a\right)+\alpha \delta_{t}e_{t}\left(s,a\right), \end{array}$$

where *δ*_*t*_ = *r*_*t*+1_ + *γQ*_*t*_(*s*_*t*+1_, *a*_*t*+1_) − *Q*_*t*_(*s*_*t*_, *a*_*t*_) and

(6) $$\begin{array}{c} e_{t}\left(s,a\right)=\{\begin{array}{cc} \gamma \lambda {e_{t}}_{-1}\left(s,a\right)+1 & \text{if}\;\;s=s_{t},\;\;a=a_{t}\\ \gamma \lambda {e_{t}}_{-1}\left(s,a\right) & \text{otherwise}. \end{array} \end{array}$$

## 5. Sarsa(*λ*)-Based Traffic Control Model

In the transport network, maximizing traffic flow and minimizing the average waiting time is the goal of scheduling and control. In traffic scheduling, junctions compete with other junctions fighting for larger traffic flow. During the course, junctions form a policy of coordination as well as constraints for adjacent junctions to maximize their own interests. Considering dynamic characteristics of the actual traffic environment, reinforcement learning algorithm based traffic control approach can be applied to get optimal scheduling policy.

**Figure 3:**
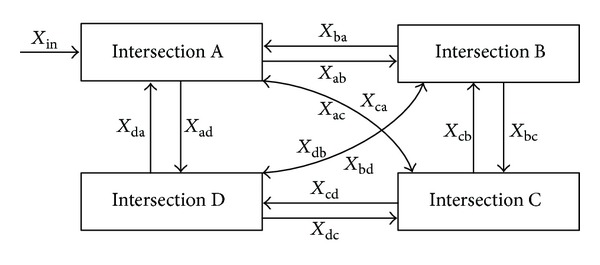
Traffic flow and control of four intersections with twelve flow directions.

In practical environment, traffic flows of four-intersections with twelve flow directions are very complex. As shown in Figure 3, there are altogether four intersections: I_a_, I_b_, I_c_, and I_d_, where

 
*X*_in_ is the intersection saturation of vehicle to intersection I_a_,
 
*X*_ab_ is the intersection saturation from intersection I_a_ to intersection I_b_,
 
*X*_ac_ is the intersection saturation from intersection I_a_ to intersection I_c_,
 
*X*_ad_ is the intersection saturation from intersection I_a_ to intersection I_d_,
 
*X*_ba_ is the intersection saturation from intersection I_b_ to intersection I_a_,
 
*X*_bc_ is the intersection saturation from intersection I_b_ to intersection I_c_,
 
*X*_bd_ is the intersection saturation from intersection I_b_ to intersection I_d_,
 
*X*_ca_ is the intersection saturation from intersection I_c_ to intersection I_a_,
 
*X*_cb_ is the intersection saturation from intersection I_c_ to intersection I_b_,
 
*X*_cd_ is the intersection saturation from intersection I_c_ to intersection I_d_,
 
*X*_da_ is the intersection saturation from intersection I_d_ to intersection I_a_,
 
*X*_db_ is the intersection saturation from intersection I_d_ to intersection I_b_,
 
*X*_dc_ is the intersection saturation from intersection I_d_ to intersection I_c_.

The control coordination between the intersections can be viewed as a Markov process, denoted by 〈*S*, *A*, *R*〉, where *S* represents the state of the intersection, *A* stands for the action for traffic control, and *R* indicates the return attained by the control agent.

### 5.1. Definition of State

Agent gets real-time traffic state and then returns traffic control decision by current state of the road. Some most important data such as intersection saturation and vehicle speed are used to reflect the state of road traffic.

Nevertheless, the traffic state is continuous, although reinforcement learning being capable of handling continuous state [21, 23] tends to make the model more complex. To simplify the algorithm, we hereby discretise saturation state and vehicle speed. The discrete saturation and speed values are shown in Table 1.

Hereby, we can obtain altogether 49 possible states by combining 7 saturations and 7 speed ranges (7∗7 = 49).

### 5.2. Definition of Action

In reinforcement learning framework, policy defines the learning agent behaviour at a given time. It in fact is a mapping from perceived states to available actions. Reinforcement learning model obtains rewards by mapping the scene to the action which affects not only the direct rewards but also the next scene, so that all subsequent rewards will be influenced. Specific states and actions are very different in various applications.

In general, traffic lights control contains five major adjustment modes: increasing green signal light duration, reducing green signal light duration, extending the signal cycle; shortening the signal light cycle, and setting all lights to red. In our study, traffic lights control actions can be categorized to 6 types: keeping the signal lights unchanged in stopping signal lights timing, extending the signal lights duration, shortening signal lights duration, setting signal lights to yellow, and setting signal lights to red. Each of them is for one of the following actual traffic scenarios.

The policy keeping the signal lights unchanged is used in the case of the normal traffic flow when the lights control strategies do not change.

The policy stopping signal lights timing is used when the traffic one direction is blocked while traffic on the other direction is normal. The policy is the last resort to release one direction traffic jam.

The policy extending the signal duration is mainly used in the case that in one direction traffic flow is blocked and the other direction is normal. Extending the signal duration increases the traffic flow while signal lights are still timing.

The policy shortening signal duration is mainly used in the case that in one direction of traffic flow is small while that of the other direction is large. Reducing signal light duration shortens the waiting time of the other direction and lets vehicles of that direction pass the intersection sooner, while signal lights keep timing.

The policy setting all lights to yellow is used for warning vehicles to slow down and keep watch.

The policy setting all lights to red is to let all the vehicles pass and clear the intersection. This policy is usually used only in emergency or the whole area is badly blocked.

In short, the action and the corresponding value are shown in Table 2.

### 5.3. Definitions of Reward and Return

Reward function in reinforcement learning defines the goal of the problem. The perceived state of the environment is mapped to a value, reward, representing internal needs of the state. The ultimate goal of reinforcement learning agent is to maximize the total reward in long term.

In our work, agent makes signal control decisions under different traffic conditions and returns an action sequence, so that by the actions the road traffic blocking indicator is the minimum. To be further, the model gives out an optimal traffic coordination mode in a certain traffic state. Here, we use traffic cost indicator to evaluate the traffic flows as

(7) $$\begin{array}{c} \text{Cost}=\omega_{1}D+\omega_{2}W, \end{array}$$

where *ω* is a weight value, *D* denotes the average delay time, and *W* represents the number of waiting vehicles.

### 5.4. Sarsa(*λ*)-Based Traffic Control Optimization

Sarsa(*λ*) learns from the original experience without environment dynamic model; it can obtain experience by interacting with environment with minimum amount of calculation cost experience; and it is a general learning model for a long-term prediction of the dynamic system. Hence we can conclude that Sarsa(*λ*) is very suitable for the real-time traffic signal control model. Hereby, we propose an on-line Sarsa(*λ*)-based traffic signal light optimization model, which overcomes the drawbacks of off-line model such as being unable to dealing with complexity and changeability of traffic control system, and requiring some accurate mathematical models. See Algorithm 1.

## 6. Simulation Experiment and Results

**Figure 4:**
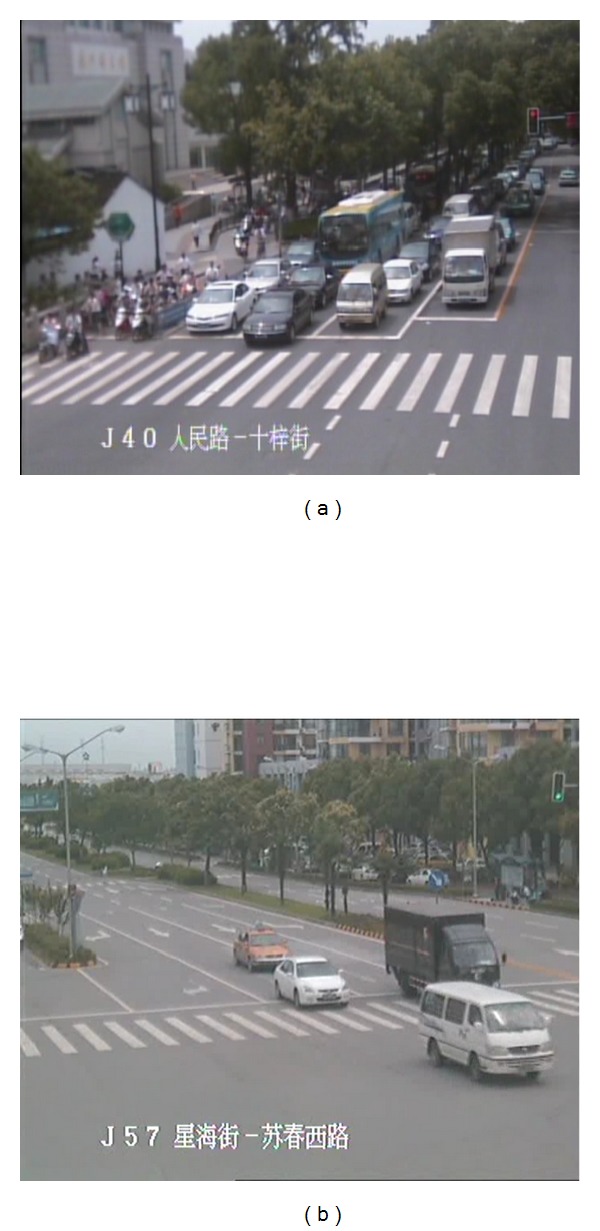
Snapshots from traffic monitoring system. (a) is snapshot of traffic in center of the city which has a heavy traffic flow and (b) is that of new distinct which has a less traffic flow.

To comprehensively evaluate behaviour of the model, we carried on simulation experiments with two different scenarios: one took advantage of an on-line traffic control optimization model and the other utilized an off-line traffic control optimization model. We also did simulation experiments in two different kinds of intersections: the city centre with heavy traffic flow and new distinct of the city with light traffic flow, as shown in Figure 4.

We utilize Sarsa(*λ*) in our study to learn a controller with learning rate = 0.5, discount rate = 0.9, and *λ* = 0.6. During learning process, cost was updated 1000 with 6000 episodes. Simulation experiments results in different intersections with an on-line control model and with an off-line control model are showed in Table 3.

We can see from Table 3 that the results optimized by the model in new distinction of the city overwhelmingly won those with an off-line optimization model; while in centre of the city, although improved, the margin of the two approaches is narrow. It is mainly because the roads of new distinction have high traffic capacity and traffic flow there is relatively smaller while the traffic flow in the centre of the city is too heavy for any intelligent model to improve.

## 7. Conclusions

Because traffic control system is so complex and changeable that an off-line traffic control model with predefined strategy can hardly cope with the traffic congestion and sudden traffic accidents which actually may occur at any time, the demand for combining timely and intelligent traffic control policy with real-time road traffic is getting more and more urgent.

Reinforcement learning accumulates experiment and knowledge by keeping interaction with environment. Although it usually needs a long duration to complete learning, it has pretty good learning ability to complex system, enabling it to handle unknown complex states well. The application of reinforcement learning in traffic management area is gradually receiving more and more concerns.

In this work, we, under the framework of reinforcement learning, propose a Sarsa(*λ*)-based learning algorithm for traffic control optimization. The actual continuous traffic states are discretized for the purpose of simplification. We design actions for traffic control and define reward and return by mean of traffic cost which combines with multiple traffic capacity indicators.

In the simulation testing experiment, we evaluated the behavior of traffic control with optimization in new distinct of the city as well as in the centre of the city. The results of traffic control optimized by our proposed on-line model were better than those optimized by off-line model.

## Figures and Tables

**Algorithm 1:**
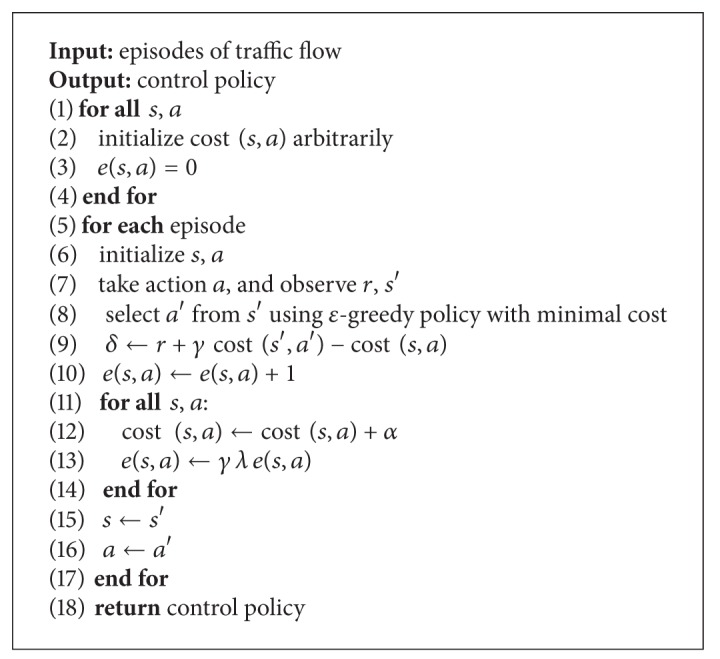
Sarsa(*λ*)-based traffic control optimization.

**Table 1:** Discrete saturation and speed values.

Saturation	Discrete value	Speed range (m/h)	Discrete value
0	0	0	0
0.1	1	(0,10]	1
0.2	2	(10,20]	2
0.3	3	(20,30]	3
0.4	4	(30,40]	4
0.5	5	(40,50]	5
0.6	6	(50,60]	6
0.7	7	>60	7

**Table 2:** Action and corresponding value.

Value	Action
1	Keeping the signal lights unchanged
2	Stopping signal lights timing
3	Extending the signal lights duration
4	Shortening signal lights duration
5	Setting signal lights to yellow
6	Setting signal lights to red

**Table 3:** Simulation experiments results in different intersection with an on-line control model and with an off-line control model.

Intersection	Scenario	Average delay time (s)	Average number of waiting vehicles
City centre	with on-line control	51.2	7.42
	with off-line control	59.1	7.63

New distinct	with on-line control	21.6	4.21
	with off-line control	39.5	5.89
